# Benzothiazole—An Antifungal Compound Derived from Medicinal Mushroom *Ganoderma lucidum* against Mango Anthracnose Pathogen *Colletotrichum gloeosporioides* (Penz and (Sacc.))

**DOI:** 10.3390/molecules28062476

**Published:** 2023-03-08

**Authors:** Gayathri Muniyappan, Thiribhuvanamala Gurudevan, Praveen Thangaraj, Akshaya Subbaih Balamurali, Arumuka Pravin Iyadurai, Rajamanickam Suppaiah, Krishnamoorthy Akkanna Subbiah, Haripriya Shanmugam

**Affiliations:** 1 Department of Plant Pathology, Tamil Nadu Agricultural University, Coimbatore 641003, India; 2 Department of Nanoscience and Technology, Tamil Nadu Agricultural University, Coimbatore 641003, India

**Keywords:** *G. lucidum*, anthracnose, benzothiazole, sporulation behaviour, mycelial inhibition

## Abstract

The present investigation is focused on exploring the possibilities of identifying biomolecules from the fruiting body of the medicinal mushroom *Ganoderma lucidum* against the mango anthracnose pathogen *Colletotrichum gloeosporioides*. The fruiting body (cap and stipe portion) of *G. lucidum* extracted with ethyl acetate solvent at a maximum inhibitory concentration of 1 percent exhibited the maximum mycelial growth inhibition of *C. gloeosporioides* with 70.10 percent and 40.77 percent, respectively. Furthermore, subjecting the ethyl acetate extracts from the cap portion of *G. lucidum* through thin layer chromatography (TLC) revealed the presence of two bands with Rf values of 0.38 and 0.35. The compounds eluted from band 1 recorded with the maximum mycelial growth inhibition of *C. gloeosporioides* by 53.77 percent followed by band 2 (46.33 percent) using an agar well diffusion test. Similarly, the analysis of ethyl acetate extracts from the cap portion of *G. lucidum* through Gas Chromatography-Mass spectroscopy (GC-MS) revealed the presence of the organoheterocyclic compound benzothiazole, as expressed in the highest peak area at 22.03 RT with the highest probability percentage (97%). Confirmation of the antifungal nature of benzothiazole was obtained by testing the standard sample of benzothiazole which showed a cent percent of inhibition on mycelial growth of *C. gloeosporioides* at 50 ppm minimum fungicidal concentration. Furthermore, benzothiazole caused abnormality in the mycelial structures, viz., distortion, shrinkage, clumping of mycelium, conidial malformation, and complete arrestment of conidial germination of *C. gloeosporioides* as observed through Scanning Electron Microscopy. The research on biomolecular extract of *G. lucidum* could be a novel and interesting concept for the possibility in suppression of plant pathogenic microbes in the natural field.

## 1. Introduction

Mango is an ancient fruit crop known around the world for its high nutritive value, superb flavor, and delicious taste. Moreover, it is a highly nourishing fruit containing carbohydrates; proteins; fats; minerals such as potassium, magnesium, sodium, and phosphorus [1]; and vitamins including vitamin A (beta carotene), vitamin B1, vitamin B2, and vitamin C (ascorbic acid). Though this fruit crop is affected by various pathogens such as bacteria, fungi, viruses, and phytoplasmas. Mango anthracnose caused by *C. gloeosporioides* (Penz) is considered to be the most devastating disease given that it can present pre-harvest as well as post-harvest and can result in yield losses of even up to 100 percent [2,3]. Under the current scenario, continuous and judicious application of fungicide against the pathogen has resulted in environmental pollution and health problems. Apart from that, it also leads to buildup of fungicide resistance in the pathogen. This situation has forced scientists to develop a fungicidal formulation from various natural sources that might provide a method to tackle the problem. In this context, macro-basidiomycete fungi are in the limelight and evoked interest globally for their bioactive compounds which have been found to have pharmaceutical and therapeutical values as well as nutritional value [4]. Moreover, mushrooms possess natural compounds exhibiting bioactivities of antifungal, antibacterial, and antiviral properties [5]. Based on the investigation, the current research is focused on the exploitation of antimicrobial compounds from *G. lucidum* against plant pathogens, as is evidenced by the invention of the fungicide Azoxystrobin from mushroom fungi *Strobilurus tenacellus.* This fungicide is effective against both downy mildew as well as powdery mildew of grapes. *Cordyceps sobolifera* has been used as a potential biocontrol agent against *Colletotrichum gleosporioides* [6]. Similarly, the report showed that a solvent extracted antimicrobial properties of various *Ganoderma* species, including *Ganoderma lucidum*, *Ganoderma applanatum,* and *Ganoderma austral,* against plant pathogenic fungi [7]. However, the authors reported that the ethyl acetate fraction of the fruiting body of ectomycorrhizal fungus *Pisolinthus tinctorius* possessed antifungal activity against soil-borne plant pathogens [8]. Similarly, the antimicrobial activity of crude culture filtrate from *L. edodes*, *G. lucidum*, *C. sinensis,* and *A. polytricha* showed maximum inhibitory activity against the mycelial growth of *C. capsici* by agar well diffusion assay [9]. Interestingly, in another study [10], the methanolic extract from fruiting bodies of *Coprinus comatus* inhibited the mycelial growth of *Fusarium* spp. The chloroform extract of antimicrobials from the fruiting body of *G. lucidum* exhibited higher antagonistic activity against *Colletotrichum capsici* [10]. In this aspect, the present study was framed to screen the antimicrobial activity of *G. lucidum* against *C. gloeosporioides,* which causes anthracnose disease in mango, and also to characterize the antimicrobial compounds which help us to develop fungicidal formulations from naturally available compounds.

## 2. Results

### 2.1. Testing the Ethyl Acetate Extracted Antimicrobial Compound from Fruiting Body (Cap and Stipe) of G. lucidum against the C. gloeosporioides

**Figure 1:**
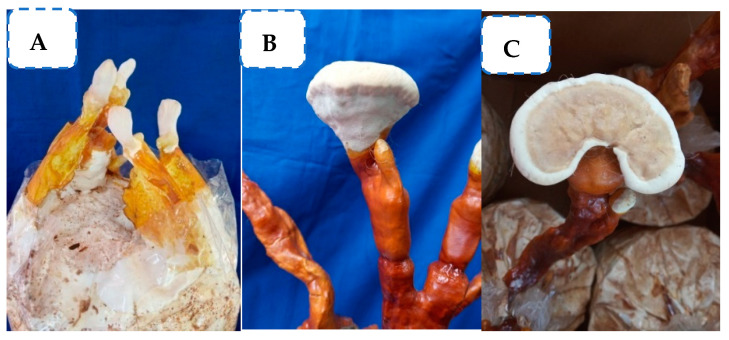
Production of the fruiting body of *G. lucidum.* (**A**) Elongation of stipe; (**B**) expansion of fruiting body; and (**C**) bracket-shaped fruiting body.

Perusal of literature revealed that the fruiting body of mushrooms is also known to possess copious antimicrobial properties. The current study was intended to tap the antimicrobial compound from the fruiting body of *G. lucidum.* For this purpose, the required fruiting bodies of *G. lucidum* were produced at the Department of Plant Pathology, Coimbatore, (image shown) by following the cultivation technology provided by the Directorate of Mushroom Research, Solan, Himachal Pradesh (www.nrcm.org.in (access on 10 September 2019)) (Figure 1). The cap and stipe portion of *G. lucidum* fruiting bodies were separated and stored for further studies.

**Figure 2:**
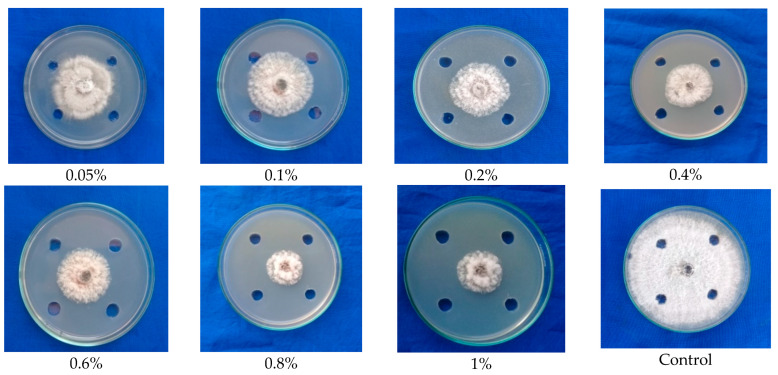
Testing the ethyl acetate-extracted antimicrobial compounds from fruiting body (cap) of *G. lucidum* against mycelial growth of *C. gloeosporioides*.

Using ethyl acetate solvent, the antimicrobial compounds were extracted from both the cap and stipe portion of the fruiting body. Furthermore, the compounds were made into different concentrations, viz., 0.05 percent, 0.1 percent, 0.2 percent, 0.4 percent, 0.6 percent, 0.8 percent, and 1 percent concentrations, to test the appropriate concentration that could inhibit the maximum mycelial growth of *C. gloeosporioides*. The result showed that the solvent (ethyl acetate) fractions of the cap portion of *G. lucidum* exhibited antifungal activity at all concentrations, as shown by mycelial inhibition ranging from 42.2 to 70.10 percent. However, maximum mycelial inhibition percentage of 70.10 was observed at a 1 percent concentration of ethyl acetate fraction of the cap portion of *G. lucidum* (Figure 2; Table 1).

**Figure 3:**
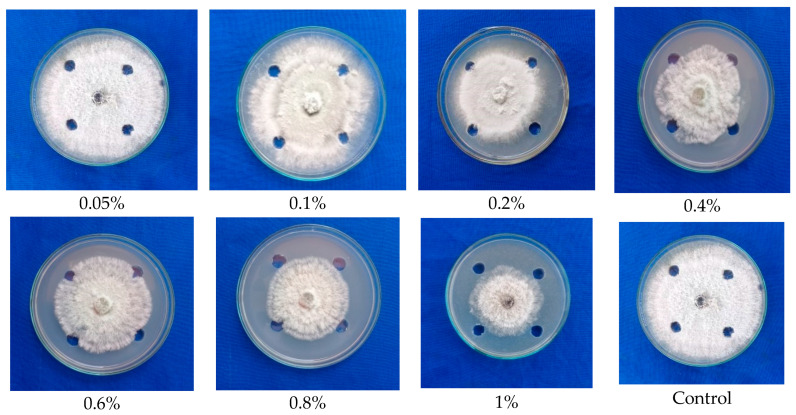
Testing the ethyl acetate extracted antimicrobial compounds from the fruiting body (Stipe) of *G. lucidum* against mycelial growth of *C. gloeosporioides*.

The solvent fraction of the stipe portion of *G. lucidum* exhibited an inhibition of mycelial growth of 34.00 percent to 40.77 percent. However, a maximum inhibition of mycelial growth of 40.77 percent was observed at 1 percent concentration (Figure 3), which was comparatively lower than the antifungal activity of fraction from the cap portion of *G. lucidum.* From the result, it is obvious that the cap portion of the fruiting body possesses maximum antimicrobial activity, and it was further subjected to GC-MS analyses to identify the compound.

### 2.2. Identifying the Presence of Antifungal Biomolecules from Ethyl Acetate Solvent Extracts of Fruiting Body (Cap) of G. lucidum through TLC Studies

**Figure 4:**
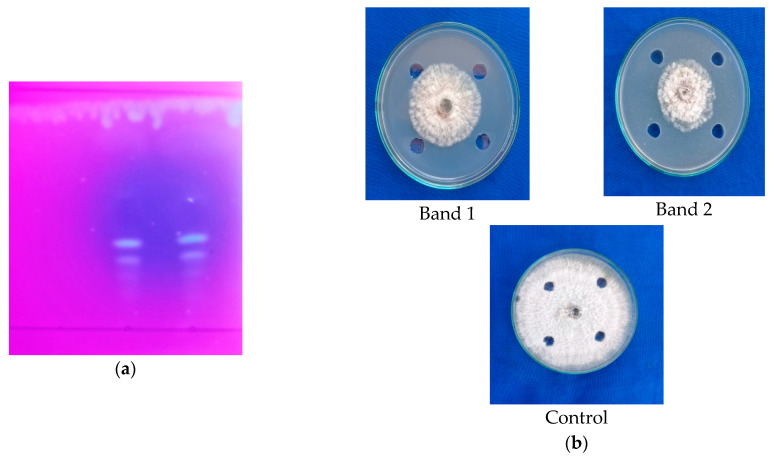
(**a**) Detection of antimicrobial compounds by ethyl acetate extracts from the fruiting body of *G. lucidum* (cap) through TLC; (**b**) antimicrobial activity of compound eluted from TLC.

Detection of specific compounds from ethyl acetate solvent extracts of the fruiting body of *G. lucidum* culture filtrates was analyzed through TLC. The TLC analysis for the ethyl acetate solvent extracts of the fruiting body (cap) of *G. lucidum* using the mobile phase Chloroform: Methanol: Water at the proportion 30:4:1 indicated the appearance of two bright bands under UV lamp at the *Rf* values of 0.38 and 0.35 (Figure 4a).

Further, the bands (band 1 and band 2) were eluted. The antifungal activity of eluted extract from the bands was tested against *C. gloeosporioides* using the agar well diffusion technique indicated that bands 1 and 2 (R_f_ value: 0.38 and 0.35, respectively) showed inhibition of mycelial growth of *C. gloeosporioides* in the range of 46.33 percent and 53.77 percent, respectively (Figure 4b; Table 2). Based on the result, it was obvious that band 2 possessed maximum antifungal activity against *C. gloeosporioides* when compared to band 1. This further confirmed that the fruiting bodies (cap) of *G. lucidum* possess higher antimicrobial activity.

### 2.3. Detection of Antimicrobial Compound from Cap Portion of Fruiting Body of G. lucidum through GC-MS

From the previous result, it was predicted that the cap portion contains more antimicrobial compounds when compared to stipe portion as observed by a mycelial inhibition of 70.10 percent. Hence, the antimicrobial compound from the cap portion of the fruiting body was separately characterized through GC-MS analysis. GC-MS analysis of ethyl acetate fraction of the fruiting body (the cap portion) indicated the presence of different compounds, viz., 5H-Cyclopropa [3,4] Benz [1,2-e] azulen-5-one; Diisooctyl phthalate; Docosanoic acid, 1,2,3-propanetriyl ester; Propanoic acid; and 7, 8-Epoxylanostan-11-ol,3-acetoxy, with most of the compounds possessing antimicrobial activity. The benzothiazole compound detected at 22.03 RT showed the highest peak area with antimicrobial activity and was used for further testing (Table 3 and Supplemental Figure S1).

### 2.4. Testing the Antifungal Activity of Standard Sample of Benzothiazole against C. gloeosporioides

From the GC-MS analysis, the compound benzothiazole from the fruiting body (cap) of *G. lucidum* was identified with high peak area percentage (22.03), as observed in a previous study. Hence, the standard sample of benzothiazole was purchased from Sigma–Aldrich chemicals (Compound code: 101338-500G) and tested against mycelial inhibition and spore germination activity of *C. gloeosporioides*.

#### 2.4.1. Testing Antifungal Activity of Benzothiazole (Standard Sample) at Different Concentrations against Mycelial Growth of *C. gloeosporioides* (Agar Well Diffusion)

Initially, the standard sample benzothiazole was dissolved in acetone, and then it was made into different inhibitory concentrations, viz., 1, 5, 10, 50, 75, 100, and 150 ppm. Furthermore, the different concentrations were tested separately against the mycelial growth of *C. gloeosporioides* via the agar well diffusion technique.

**Figure 5:**
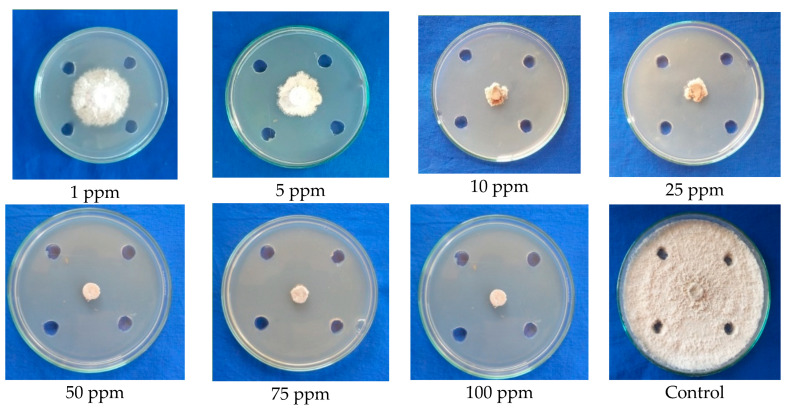
Conformation of the antifungal efficacy of benzothiazole at different concentrations against mycelial growth of *C. gloeosporioides* (Agar well test).

Based on the result obtained, it was found that the compound benzothiazole completely arrested the mycelial growth at the maximum inhibitory concentration of 50 ppm. The compound showed 50 percent mycelial inhibition at 1 ppm MIC. However, the growth pattern of mycelium in Petri plate was observed as the push back of mycelium near the agar well zone, deformation of mycelium, blackened mycelial growth, and breakage of mycelial disc; whereas the control showed normal mycelial growth with concentric rings (Figure 5; Table 4).

#### 2.4.2. Confirming the Antifungal Activity of Benzothiazole (Standard Sample) against Mycelial Growth of *C. gloeosporioides* (Paper Disc Assay)

**Figure 6:**
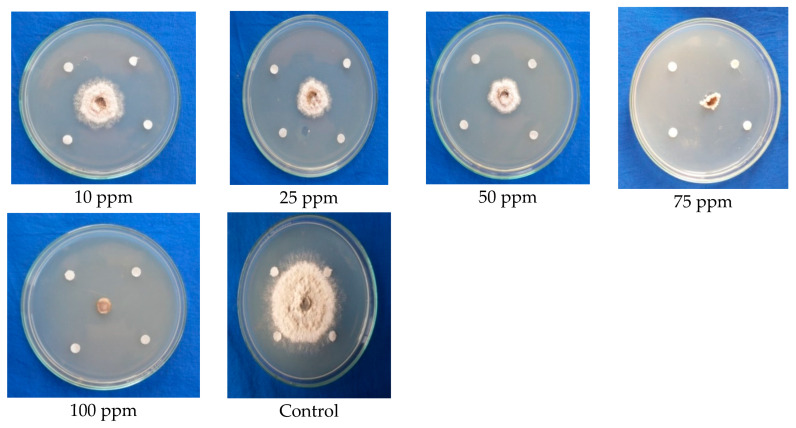
Confirmation of the antifungal efficacy of benzothiazole at different concentrations against mycelial growth of *C. gloeosporioides* (paper disc assay).

The antifungal activity of benzothiazole (standard sample) was further cross-checked via a paper disc assay. The result revealed that benzothiazole was also found to exhibit 100 percent inhibition at the maximum fungicidal concentration of 75 ppm in the paper disc assay. It clearly depicted that the compound has remarkably higher antifungal activity against *C. gloeosporioides*, as observed by the linear mycelium growth towards the paper disc, changes in the growth pattern of mycelium, retardation of mycelium growth, shrinkage of mycelial growth, and complete arresting of mycelial growth when compared to the control with normal mycelial growth (Figure 6; Table 5).

#### 2.4.3. Testing the Effect of Benzothiazole (Standard Sample) at Optimum Concentration (50 ppm) on Conidial Germination of *C. gloeosporioides*

Antifungal activity of benzothiazole was tested against the conidial germination of the target pathogen by the cavity slide method. The result revealed that no conidial germination was observed at 6 h in both the control and the treated groups. However, the conidial germination was 26.82 numbers in the control at 12 h and 50.34 numbers at 24 h; whereas the benzothiazole completely reduced the conidial germination of *C. gloeosporioides,* and no germination was observed even at 24 h. The compound showed 100 percent inhibition of conidial germination at 6, 12, and 24 h compared to the control (Table 6).

### 2.5. Antifungal Effect of Benzothiazole (Standard Sample) against Spores and Mycelium of C. gloeosporioides by Scanning Electron Microscopy (SEM)

**Figure 7:**
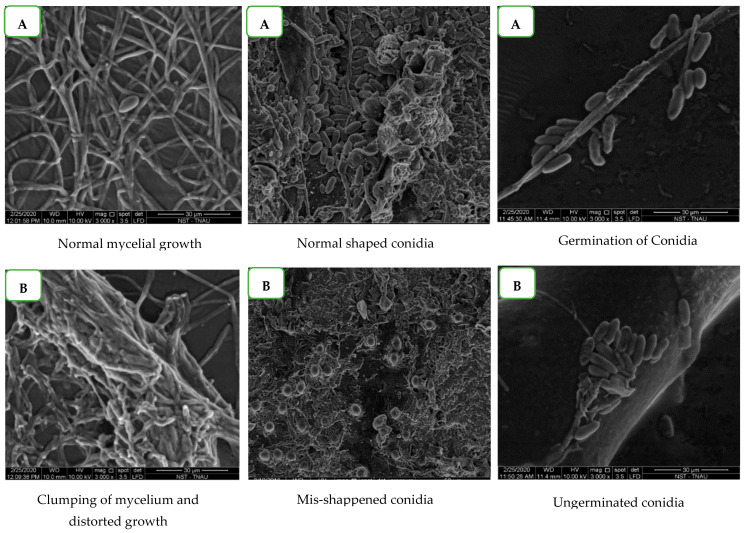
Effect of benzothiazole on germination of spores of *C. gloeosporioides* under Scanning Electron Microscopy (SEM). (**A**) Control. (**B**) Treatment.

The antifungal activity of benzothiazole on the morphological changes of mycelium and conidia of *C. gloeosporioides* was observed under Scanning Electron Microspcopy (SEM). The results showed that the standard sample benzothiazole completely inhibited the conidial germination of *C. gloeosporioides* when compared to the control (untreated), in which a certain percentage of conidial germination was observed (Figure 7). Further, distortion, shrinkage, clumping of mycelium, and conidial malformations were prominent in the benzothiazole treated samples. The untreated mycelium and conidia were normal without any morphological changes. The results further clearly depicted the antifungal nature of benzothiazole, which proves that *G. lucidum* possess biomolecules with fungicidal activity.

## 3. Discussion

Mushrooms contain various natural compounds with bioactive potential, viz., antifungal, antibacterial, antiviral, antitumor, anti-nemic, anti-inflammatory, anti-allergic, anti-atherogenic, and anti-diabetic properties. Every species of mushrooms contains various bioactive molecules and pharmacological effects [11]. Hence, it was decided to investigate the antimicrobial compound from the fruiting body of *G. lucidum,* including both the cap and stipe portions separately against *C. gloeosporioides*.

The result showed that the solvent (ethyl acetate) fractions of the cap portion of *G. lucidum* exhibited antifungal activity ranging from 42.2 to 70.10 percent at all concentrations. However, a maximum mycelial inhibition percentage of 70.10 was observed at a 1 percent concentration of ethyl acetate fraction of the cap portion of *G. lucidum.* The solvent fraction of the stipe portion of *G. lucidum* which exhibited maximum inhibition of mycelial growth of 40.77 percent was at 1 percent concentration, which was comparatively lower than the antifungal activity of the fraction from the cap portion of *G. lucidum*. Our findings were in line with the scientist who tested the solvent-extracted antimicrobial property of various *Ganoderma* species, including *G. lucidum*, *G. applanatum* and *G. austral,* against plant pathogenic fungi and found that the crude extract of *G. lucidum* showed a greater zone of inhibition (24.3 mm) against *Aspergillus niger* followed by *G. applanatum* (23.3 mm) [7].

Detection of specific compounds from ethyl acetate solvent extracts of the fruiting body of *G. lucidum* culture filtrates was analyzed through TLC, which indicated the appearance of two bright bands under a UV lamp at the Rf values of 0.38 and 0.35. Furthermore, the bands (band 1 and band 2) were eluted, and their antifungal activity was tested against *C. gloeosporioides* using the agar well diffusion technique. The results indicated that bands 1 and 2 (Rf values of 0.38 and 0.35, respectively) eluted from the TLC plate showed inhibition of mycelial growth of *C. gloeosporioides* in the range of 46.33 percent and 53.77 percent, respectively. This further confirmed that the fruiting bodies (cap) of *G. lucidum* possess higher antimicrobial activity. Accordingly, [10] performed the TLC analysis for the chloroform extracts of the fruiting body of *G. lucidum*, and the results indicated the presence of two bands with Rf values of 0.29 and 0.62. The eluted compounds showed inhibition percentages of 66.67 and 60.42 against the mycelial growth of *C. capsici.*

In the present study, the GC-MS analysis of the ethyl acetate fraction of the fruiting body (the cap portion) of *G. lucidum* indicated the presence of different compounds, viz., 5H-Cyclopropa [3,4] Benz[1,2-e] azulen-5-one; Diisooctyl phthalate; Docosanoic acid, 1,2,3-propanetriyl ester; Propanoic acid; and 7,8-Epoxylanostan-11-ol, 3-acetoxy, with most of the compounds possessing antimicrobial activity. H-Cyclopropa [3,4] Benz[1,2-e] azulen-5-one possessed antibacterial property. Propanoic acid and benzothiazole possessed antifungal activity, whereas 7,8-Epoxylanostan-11-ol, 3-acetoxy possessed anti-nemic activity. The organoheterocyclic compound benzothiazole, a compound detected at 22.03 RT, showed the highest peak area with antimicrobial activity and was used for further studies. Accordingly, [12] identified bioactive compounds including triterpenoid and vulpinic acid from the fruiting body of *Scleroderma citrinum,* which showed antagonistic activity against mycelial growth of *Candida albicans*. This is in line with the findings [13] from which they also reported that the compounds obtained from the fruiting body of *Scleroderms citrinum,* including 4,4-Dimethoxy methyl vulpinate (DMV) and 4,4 Dimethoxy vulpinic acid (DMVA), were found to possess antifungal activity against *C. gloeosporioides*. Another report [8] showed that three olypins such as 9-hydroxy 10, 12-octadecadienoic acid; 13-oxo 10, 12 octadecadienoic acid; and 9-oxo 10, 12 octadecadienoic acid from *Pisolithus tinctorius* showed antagonistic activity against plant pathogenic fungi such as *C. gloeosporioides*, *C. fragariae*, *C. acutatum*, *Botrytis cinerea*, *F. oxysporum*, *Phomopsis obscurang,* and *Phomopsis viticola.*

Benzothiazole is an aromatic organoheterocyclic compound and also a potent antimicrobial secondary metabolite and a volatile compound found in plants and bacteria. On the other hand, the mechanism of benzothiazole against plant pathogens is still unclear. Here, in this study, a complete mycelial inhibition was observed at 50 ppm concentration of benzothiazole, and a 50 percent mycelial inhibition was observed at 1 ppm concentration. The growth pattern of mycelium was observed as mycelium pushed back near the agar well zone, distortion in mycelial growth, blackening of mycelial growth, and breakage of the mycelial disc; whereas the control showed normal mycelial growth with concentric rings. Remarkably, the benzothiazole compound possessed antimicrobial activity against *Phytophthora capsica,* disrupted the cell membrane, and inhibited the hyphal growth of the fungi [14]. More fascinatingly, benzothiazole possessed antifungal activity against *Penicillium italicum,* which causes postharvest navel orange. Furthermore, it acts as the analog of naturally occurring salicylic acid and is highly effective for induction of systemic acquired resistance (SAR) in plants. These findings line up with our finding, namely that benzothiazole possesses antifungal activity against *C. gloeosporioides*.

The results observed under SEM showed that the standard sample of benzothiazole completely inhibited the conidial germination of *C. gloeosporioides* when compared to the control (untreated), in which conidial germination was observed. Furthermore, distortion, shrinkage, clumping of mycelium, and conidial malformations were prominent in the benzothiazole treated samples. The untreated mycelium and conidia were normal without any morphological changes. The results further depicted the antifungal nature of benzothiazole, which proves that *G. lucidum* possesses biomolecules with fungicidal activity. Furthermore, the results clearly depict the antisporulant nature of benzothiazole. Similar to our study, [14] observed the hyphal morphology of *Phytophthora capsici* treated with benzothiazole concentration 150 mg/L at different hours of incubation and showed that the hyphae treated with benzothiazole exhibited swelling and contortion at 24 h. The hyphae produced more abnormal branches after exposure for 48 h, and the hyphae began to develop periodical swellings after exposure for 72 h.

## 4. Material and Methods

### 4.1. Production of the Fruiting Body of G. lucidum

Based on the protocol (www.dmr.org.in (accessed on 10 September 2019)) provided by the Directorate of Mushroom Research, Solan, the cultivation of the fruiting body of *G. lucidum* was carried out at the Department of Plant Pathology, Tamil Nadu Agricultural University, Coimbatore, India. Coconut sawdust along with 20% wheat bran was used as a substrate for mushroom production. Optimum moisture of 65% was maintained in sawdust, and it was packed at 1 kg in a polypropylene bag plugged with non-absorbent cotton. The bag was sterilized using autoclave at 121 °C, 15 lbs for 2 h. After autoclaving, the sterilized bags were spawned (2%) with the sorghum-based spawn of *G. lucidum*, and the bags were incubated at 28 ± 2 °C for the complete run of spawn run up to 35 days. Furthermore, the bags were transferred into the cropping room, and the optimum temperature of 28 °C and 80% RH were maintained for the emergence of a pinhead. Later, the temperature was lowered to 25 °C with 60% RH for cap maturation. Required quantities of fruiting bodies were harvested and kept for further analysis.

### 4.2. Extraction of Antimicrobial Compounds from Fruiting Body of G. lucidum

The fruiting body of *G. lucidum* was harvested, and the cap and stipe portion were separated. The cap and stipe portion were ground separately into a fine powder using a pestle and mortar. For 10 g of the powdered sample, 100 mL of ethyl acetate solvent was added. The flasks were kept overnight in a shaker at 150 rpm. Later, they were filtered through double-layered muslin cloth followed by Whatman No. 1 filter paper, and the solvents were evaporated using a vacuum flask evaporator at 40 °C. The residue obtained was dissolved in HPLC grade methanol. The extracts were filtered through a membrane filter (0.2 µm) and stored at 4 °C for further research. The same procedure was followed for the stipe portion of the fruiting body.

#### In Vitro Antimicrobial Activity of Ethyl Acetate Extracts from Fruiting Body of *G. lucidum* against *C. gloeosporioides*

The ethyl acetate solvent extracts from the cap and stipe portions of *G. lucidum* were tested against the mycelial growth of *C. gloeosporioides* at different concentrations from 0.1 percent to 1 percent via the agar well diffusion technique. Four wells were made at equal distance, with a margin of 1 cm from the periphery of the Petri plate containing sterilized PDA medium. About 100 µL from the extracts of each concentration was added to the plate. A 9 mm mycelial disc of *C. gloeosporioides* was placed at the center of the plate. Three replications were maintained for each concentration. Sterile water served as the control. Plates were incubated at room temperature, and the percentage inhibition of mycelial growth in pathogen over the control was calculated by using the formula given in [15].

### 4.3. Identification of Antimicrobial Compounds Solvent Extracts of the Fruiting Body (Cap) of G. lucidum through TLC

Antimicrobial compounds from the ethyl acetate-extracted fruiting body (the cap portion) extracts of G. lucidum were spotted on the silica-gel-coated TLC plates at the rate of 20 µL/spot. The spots were allowed to dry, and the chromatograph (Thermostat Vertical Autoclave, STE-V 60, MRC lab, India) was developed using a solvent system, viz., Chloroform: Methanol: Distilled water 30:4:1. The plate was allowed to run at the ¾th position of the plate. Then, the plates were observed under a UV transilluminator (UV 25 Transilluminator, Hoefer Scientific, Hollistan, MA, USA) at 254 nm. Bands observed in the UV lamp were marked, and the Rf value was calculated and recorded.

#### Testing the Antifungal Activity of TLC Eluted Compounds of *G. lucidum* against *C. gloeosporioides*

Specific bands obtained through TLC studies were scrapped from the silica plate and suspended in 1mL HPLC grade methanol. The solution was vortexed for 10–15 min and then further centrifuged at 10,000 rpm for 10 min. The methanol along with the compound and the silica were separated and filtered through a membrane filter (0.2 µm), and the antimicrobial activity of the compound was tested against the mycelial growth of *C. gloeosporioides* via the agar well diffusion technique by following the procedure as described earlier with three replications.

### 4.4. Profiling of Antimicrobial Compounds from G. lucidum through GC-MS

Based on the results obtained from the previous study, the ethyl acetate-extracted antimicrobial compound from the cap portion of *G. lucidum* showed a quite higher mycelial inhibition percentage against *C. gloeosporioides* than the stipe portion. Therefore, the antimicrobial compound from the cap portion of the fruiting body was characterized through GC-MS analysis. In this study, the Trace GC Ultra and DSQII model MS from Thermo Fisher Scientific Limited, Markha, Canada were engaged for analysis [9,16].

### 4.5. Testing the Antifungal Activity of the Standard Sample (Benzothiazole) against C. gloeosporioides

From the GC-MS analysis, the compound benzothiazole from the fruiting body (cap) of *G. lucidum* with a high peak area and probability percentage were identified as the antimicrobial compound. Hence, to confirm the antifungal activity, the standard sample benzothiazole was purchased from Sigma-Aldrich chemicals (Sigma Aldrich, Saint Louis, MO, USA) and tested against mycelial inhibition of *C. gloeosporioides* via the agar well diffusion technique.

Benzothiazole standard sample was tested at lower concentrations because higher concentrations inhibited complete mycelial growth. Thus, the standard sample of benzothiazole was dissolved in acetone, and then it was made into different concentrations, viz., 1, 5, 10, 50, 75, and 100 ppm. Furthermore, the different concentrations were tested separately against the mycelial growth of *C. gloeosporioides* by agar well diffusion assay, as discussed earlier, with three replications for each treatment.

### 4.6. Effect of Standard Sample Benzothiazole on C. gloeosporioides under Scanning Electron Microscopy

Scanning Electron Microscope analysis was carried at the Department of Nano Science and Technology, Tamil Nadu Agricultural University, Coimbatore, and was used to assess the changes in mycelium and conidia of *C. gloeosporioides* due to the fungicidal action of benzothiazole at 50 ppm. The morphostructural changes of the pathogen were viewed under Scanning Electron Microscope (SEM: Quanta 250, FEI, Hillsboro, OR, USA) with Large Field Detector (LFD). The SEM was operated in vacuum 10 KV with a spot size of 3.0 and a pressure of 60 Pa. The sample images were recorded at 5000× magnification. The source of electrons used in the SEM was Tungston Filament, and Thermoionic emission was used for the detection of the samples in SEM.

### 4.7. Statistical Analysis

The design of experiments (CRBD) and statistical analyses were followed as per the suggestions of (Gomez and Gomez, 1984). Statistical software SPSS (version number 16.0) was used for the analyses of the data. In case of zero values, the data were arcsine-transformed (1/4n) before statistical analysis.

## 5. Conclusions

The results of the current research overwhelmingly indicated the presence of innate and novel biomolecules in the medicinal mushroom *G. lucidum,* with antimicrobials which performed well in inhibiting both vegetative and reproductive structures of *C. gloeosporioides*. The compound benzothiazole showed promising effects against *C. gloeosporioides*. Future studies are planned towards studying the mechanism of benzothiazole and its role in secretion of defense enzymes against pathogens in plants. Thus, *G. lucidum* offers a spectrum of opportunity for setting a pathway on the eco-friendly management of anthracnose disease in mango plants.

## Figures and Tables

**Table 1:** Testing the ethyl acetate-extracted antimicrobial compound from the fruiting body (cap and stipe) of *G. lucidum* against *C. gloeosporioides*.

Concentration (%)	Solvent Extracts of *G. lucidum*- Cap		Solvent Extracts of *G. lucidum*- Stipe	
	* Mean Mycelial Growth (mm)	Percent Inhibition over Control	* Mean Mycelial Growth (mm)	Percent Inhibition over Control
0.05	52.1 (46.15)	42.20 ^e^ (40.51)	90.0 (71.61)	0.00
0.1	48.3 (44.00)	46.60 ^d^ (43.05)	90.0 (71.61)	0.00
0.2	41.6 (40.14)	53.77 ^c^ (47.16)	58.6 (49.93)	34.00 ^d^ (35.65)
0.4	40.2 (39.33)	55.59 ^c^ (48.26)	57.6 (49.35)	36.10 ^bc^ (36.91)
0.6	36.0 (36.85)	60.00 ^b^ (50.77)	58.3 (49.75)	35.22 ^cd^ (36.38)
0.8	30.0 (33.19)	67.71 ^a^ (55.38)	56.3 (48.60)	37.44 ^b^ (37.71)
1.0	26.7 (31.09)	70.10 ^a^ (56.85)	53.3 (46.87)	40.77 ^a^ (39.66)
Control	90.0 (70.61)	0.0 (0.51)	90.0 (71.61)	0.00 (0.57)
SE(d)	0.515	-	1.155	-
CD (*p* = 0.05)	1.114	-	2.502	-

* Values are the mean of three replications. Means followed by a common letter are not significantly different at the 5% level by DMRT (Numerical letter indicated on percent inhibit represent the least inhibition of pathogen). Values in parenthesis are arcsine-transformed values.

**Table 2:** Testing the antimicrobial activity of compounds eluted from TLC band of *G. lucidum* (extracts from fruiting body) against mycelial growth of *C. gloeosporioides*.

Compound	* Mean Mycelial Growth (mm)	Percent Inhibition over Control
Band 1	48.3 (44.00)	46.33 ^b^ (42.87)
Band 2	41.6 (40.14)	53.77 ^a^ (47.16)
Control	90.0 (71.61)	0.0 (0.57)
SE (d)	0.562	-
CD (*p* = 0.05)	1.603	-

* Values are the mean of five replications. Means followed by a common letter are not significantly different at 5% level by DMRT test (Numerical letter indicated on percent inhibit represent the least inhibition of pathogen). Values in parenthesis are arcsine-transformed values.

**Table 3:** List of antimicrobial compounds from ethyl acetate solvent fractions from fruiting body of *G. lucidum* using GCMS.

RT	Compound	Biological Activity	Molecular Weight (g/mol)	Molecular Formula
14.16	5H-Cyclopropa[3,4]benz[1,2-e]azulen-5-one	Antibacterial anti inflammatory	506.548	C_26_H_34_O_10_
15.10	Diisooctyl phthalate	-	390.564	C_24_H_38_O_4_
17.71	Docosanoic acid, 1,2,3-propanetriyl	Emulsifying agent	1059.825	C_69_H_134_O_6_
18.42	Propanoic acid	Antifungal	74.079	C_3_H_6_O_2_
22.03	Benzothiazole	Antifungal	135.19	C_7_H_5_NS
26.29	7,8-Epoxylanostan-11-ol,3-acetoxy-	Antimicrobial	502.78	C_32_H_54_O_4_

**Table 4:** Testing the antifungal efficacy of benzothiazole at different concentrations against mycelial growth of *C. gloeosporioides*.

Conc. (ppm)	* Mean Mycelial Growth (mm)	Percent Inhibition over Control	Growth Pattern of Mycelium
1	40.10	55.44 ^c^	Mycelium pushed back near the agar well zone
	(39.27)	(48.10)	
5	35.30	60.77 ^c^	Distortion in growth pattern of mycelium
	(36.43)	(51.20)	
10	6.00	93.33 ^b^	Blackening of mycelial growth
	(14.17)	(75.29)	
25	4.20	95.53 ^b^	Distortion of mycelial growth
	(11.82)	(78.06)	
50	0.00	100.00 ^a^	Breakage in the mycelial disc
	(0.51)	(80.38)	
75	0.00	100.00 ^a^	Completely arrested mycelium
	(0.51)	(80.38)	
100	0.00	100.00 ^a^	Completely arrested mycelium
	(0.51)	(80.38)	
Control	90.0	0.00	Normal growth of mycelium with concentric rings
	(71.61)	(0.57)	
SE (d)	0.353	-	-
CD (*p* = 0.05)	0.827	-	-

* Values are the mean of three replications. Means followed by a common letter are not significantly different at 5% level by DMRT (Numerical letter indicated on percent inhibit represent the least inhibition of pathogen). Values in parenthesis are arcsine-transformed values.

**Table 5:** Testing the antifungal efficacy of different concentrations of benzothiazole against mycelial growth *C. gloeosporioides* by paper disc assay.

Minimum Fungicidal Concentration (ppm)	* Mean Mycelial Growth (mm)	Percent Inhibition over Control	Growth Pattern
10	37.1	58.77 ^b^	Linear mycelium growth towards the paper disc
	(37.50)	(50.54)	
25	35.3	60.77 ^b^	Changes in growth pattern of the mycelium
	(36.43)	(51.22)	
50	32.1	64.44 ^b^	Retardation of mycelium from the paper disc
	(34.46)	(53.39)	
75	0.00	100.00 ^a^	Shrinkage of mycelial disc
	(0.57)	(84.16)	
100	0.00	100.00 ^a^	No growth of mycelium
	(0.57)	(84.16)	
Control	70.4	0.00	Normal growth of mycelium with concentric rings
	(56.85)	(0.57)	
SE (d)	0.453	-	
CD (*p* = 0.05)	1.130	-	

* Values are the mean of three replications. Means followed by a common letter are not significantly different at 5% level by DMRT (Numerical letter indicated on percent inhibit represent the least inhibition of pathogen). Values in parenthesis are arcsine-transformed values.

**Table 6:** Testing the effect of benzothiazole (50 ppm) on germination of spores of *C. gloeosporioides*.

Time of Interval	Conidial Germination of *C. gloeosporioides* at 50 ppm		Reduction in Germination Percent over Control
	Control (No.)	Treatment (No.)	
6 h	0.00	0.00	0.00
12 h	26.82 (31.191)	0.00	100
24 h	50.34 (45.19)	0.00	100
CD (*p* = 0.05)	1.649		

Values are the mean of five replications. Values in parenthesis are arcsine-transformed values.

## Data Availability

All the data are available in the manuscript.

## Supplementary Materials

The following supporting information can be downloaded at: https://www.mdpi.com/article/10.3390/molecules28062476/s1, Figure S1: Characterization of antimicrobial compounds from ethyl acetate solvent fractions from the fruiting body of *G. lucidum.*

## Sample Availability

Samples of the compounds benzothiazole are available from the authors.
