# Supplementary material for: Benzothiazole—An Antifungal Compound Derived from Medicinal Mushroom Ganoderma lucidum against Mango Anthracnose Pathogen Colletotrichum gloeosporioides (Penz and (Sacc.))
Source: Molecules. 2023 Mar 8;28(6):2476. doi: 10.3390/molecules28062476 (PMC10053814; doi:10.3390/molecules28062476)

Figure S1. Characterization of antimicrobial compounds from Ethyl acetate solvent fractions from fruiting body of *G. lucidum*

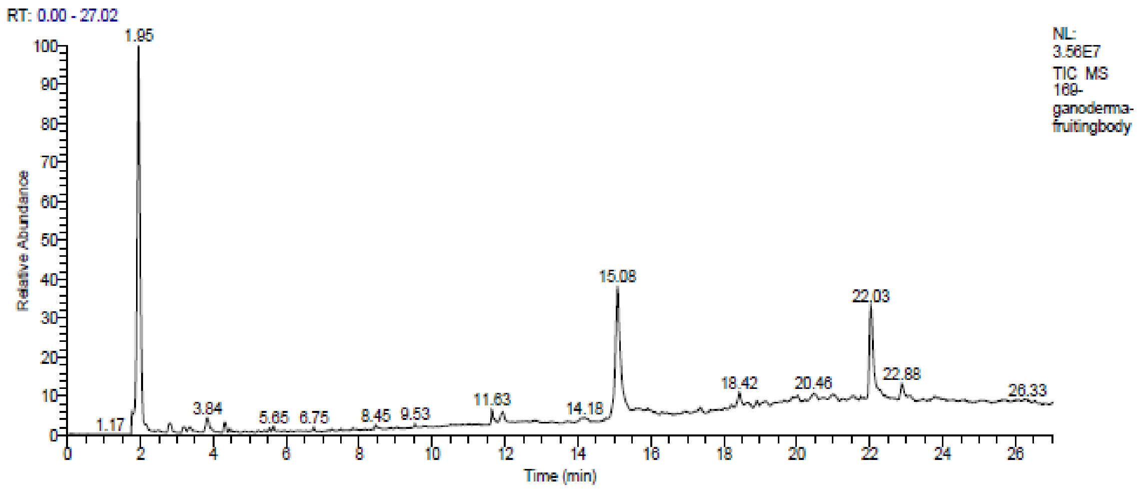

Supplement: Supplementary file 1 [file molecules-28-02476-s001.zip › molecules-2097596-supplementary.pdf]
